# Standardized image interpretation and post processing in cardiovascular magnetic resonance: Society for Cardiovascular Magnetic Resonance (SCMR) Board of Trustees Task Force on Standardized Post Processing

**DOI:** 10.1186/1532-429X-15-35

**Published:** 2013-05-01

**Authors:** Jeanette Schulz-Menger, David A Bluemke, Jens Bremerich, Scott D Flamm, Mark A Fogel, Matthias G Friedrich, Raymond J Kim, Florian von Knobelsdorff-Brenkenhoff, Christopher M Kramer, Dudley J Pennell, Sven Plein, Eike Nagel

**Affiliations:** 1 Working Group on Cardiovascular Magnetic Resonance, Experimental and Clinical Research Center, a joint cooperation between the Charité Medical Faculty and the Max-Delbrueck Center for Molecular Medicine, and HELIOS Klinikum Berlin Buch, Department of Cardiology and Nephrology, Charité Medical University Berlin, Berlin, Germany; 2 Departments of Medicine and Radiology and the Cardiovascular Imaging Center, University of Virginia Health System, Charlottesville, VA, USA; 3 Imaging, and Heart and Vascular Institutes, Cleveland Clinic, Cleveland, OH, USA; 4 Radiology and Imaging Sciences, National Institutes of Health Clinical Center, Bethesda, MD, USA; 5 Leeds Institute for Genetics Health and Therapeutics & Leeds Multidisciplinary Cardiovascular Research Centre, University of Leeds, Leeds, UK; 6 Duke Cardiovascular Magnetic Resonance Center, and Departments of Medicine and Radiology, Duke University, University Medical Center, Durham, NC, USA; 7 CMR Centre at the Montreal Heart Institute, Department of Cardiology, Université de Montréal, Montreal, Canada; 8 Royal Brompton Hospital, and Imperial College, London, UK; 9 Department of Radiology, Children’s Hospital of Philadelphia, University of Pennsylvania School of Medicine, Philadelphia, PA, USA; 10 Department of Radiology of the University Hospital Basel, Basel, Switzerland; 11 Division of Imaging Sciences and Biomedical Engineering, Department of Cardiovascular Imaging, King’s College, London, UK

**Keywords:** Magnetic resonance imaging, Heart, Recommendations, Image interpretation, Post processing

## Abstract

With mounting data on its accuracy and prognostic value, cardiovascular magnetic resonance (CMR) is becoming an increasingly important diagnostic tool with growing utility in clinical routine. Given its versatility and wide range of quantitative parameters, however, agreement on specific standards for the interpretation and post-processing of CMR studies is required to ensure consistent quality and reproducibility of CMR reports. This document addresses this need by providing consensus recommendations developed by the Task Force for Post Processing of the Society for Cardiovascular MR (SCMR). The aim of the task force is to recommend requirements and standards for image interpretation and post processing enabling qualitative and quantitative evaluation of CMR images. Furthermore, pitfalls of CMR image analysis are discussed where appropriate.

## Preamble

With mounting data on its accuracy and prognostic value, cardiovascular magnetic resonance (CMR) is becoming an increasingly important diagnostic tool with growing utility in clinical routine. Given its versatility and wide range of quantitative parameters, however, agreement on specific standards for the image interpretation and post processing of CMR studies is required to ensure consistent quality and reproducibility of CMR reports. This document addresses this need by providing consensus recommendations developed by the Task Force for Post Processing of the Society for Cardiovascular MR (SCMR). The aim of the task force is to recommend requirements and standards for image interpretation and post processing enabling qualitative and quantitative evaluation of CMR images. Furthermore, pitfalls of CMR image analysis are discussed where appropriate.

The Task Force is aware that for many of the recommendations, the body of evidence is limited. Thus, this document represents expert consensus providing guidance based on the best available evidence at present as endorsed by the SCMR. As CMR undergoes rapid development, updated recommendations for image acquisition, interpretation and post processing are needed regularly and will be provided by online appendices when needed and updated Task Force papers in due course.

The recommendations are considered for the application of CMR in clinical routine. For some applications, quantification is considered as providing added information but is not mandatory (e.g perfusion imaging), whereas for others quantification is required for all clinical reports (e.g. T2* assessment in iron overload). In general the intention of this task force is to describe, in which scenarios quantitative analysis should be performed and how it is performed.

The recommendations respect societal recommendations for structured reporting of cardiovascular imaging studies in general (ACCF / ACR / AHA / ASE / ASNC / HRS / NASCI / RSNA / SAIP / SCAI / SCCT / SCMR) [1] and in specific for CMR studies (SCMR) [2]. These recommendations will be reviewed and updated regularly and updates made available on the SCMR website.

The recommendations do not supersede clinical judgment regarding the contents of individual interpretation of imaging studies.

The Task Force made every effort to avoid conflicts of interests and, where present, to disclose potential conflicts.

## General recommendations

The recommendations listed in this section apply to the acquisition and post processing of all CMR data. CMR studies should be performed for recommended indications [3], respecting published appropriateness criteria [4] and the recently published societal CMR expert consensus document [5]. Any analysis should be performed using uncompressed or lossless compressed DICOM (*Digital Imaging and Communications in Medicine*) source images. Readers should have adequate training and clinical experience. The identity and responsibility of the reader should be appropriately documented in the report. Data acquisition should conform to the recommendations of SCMR [6].

Furthermore, the reader of clinical data is also responsible for the use of adequate post processing hardware and software. The general requirements include

– Workstation and screen of adequate specification and resolution (as per the specifications of the post-processing software)

– Post processing software with regulatory approval for use in patients, ideally providing the following tools:

– Ability to view all short-axis cines in a single display

– Ability to perform endocardial and epicardial contour tracings on short-axis cines

– Ability to correct for atrioventricular annular location from the long-axis slice onto the most basal left ventricular (LV) short-axis location in contour tracings

– Cross references for confirmation of slice position

– Ability to compare cine, late gadolinium enhancement (LGE) and/or perfusion images from the same location simultaneously

– Ability to compare short- and long-axis images of the same region simultaneously

– Ability to compare images of the approximate same location on the current and prior study simultaneously for longitudinal studies

– Ability to perform (semi-) quantitative signal intensity (SI) analysis

– Ability to perform standardized segmentation of the myocardium according to the model of the American Heart Association (AHA) [7]

– Ability to use baseline-correction or comparison to a phantom for flow measurements can be helpful

– Ability to manually correct heart rate, weight, body surface area

– Regarding evaluation of angiography the software should provide the following tools:

– Subtraction of post-contrast from pre-contrast 3D datasets

– 3D multiplanar and maximum intensity projection (MIP) capabilities

– Volume rendering and surface shaded reconstructions optional for reporting but not mandatory for quantitative analysis

– Quantitative diameter analysis based on non-subtracted 3D-MR angiography (MRA)

– MIP reconstruction based on non-subtracted or subtracted 3D-MRA datasets

## Left ventricular chamber assessment

### 1. Visual analysis

a) Before analyzing the details, review all cines in cine mode, validate observations from one plane with the others, and check for artifacts.

b) Dynamic evaluation of global LV function: Interpretation of both ventricular chambers, in concert with extracardiac structures including assessment for hemodynamic interaction between the two chambers (e.g., shunts, evidence of constrictive physiology).

c) Assessment of LV function from a global and segmental perspective. Segmental wall motion is based on segmental wall thickening during systole. Wall motion is categorized as: hyperkinetic, normokinetic, hypokinetic, akinetic, dyskinetic.

d) In presence of segmental wall motion abnormalities, use of standard LV segmentation nomenclature corresponding to the supplying coronary artery territories is recommended [2,7].

### 2. Quantitative analysis

**Figure 1:**
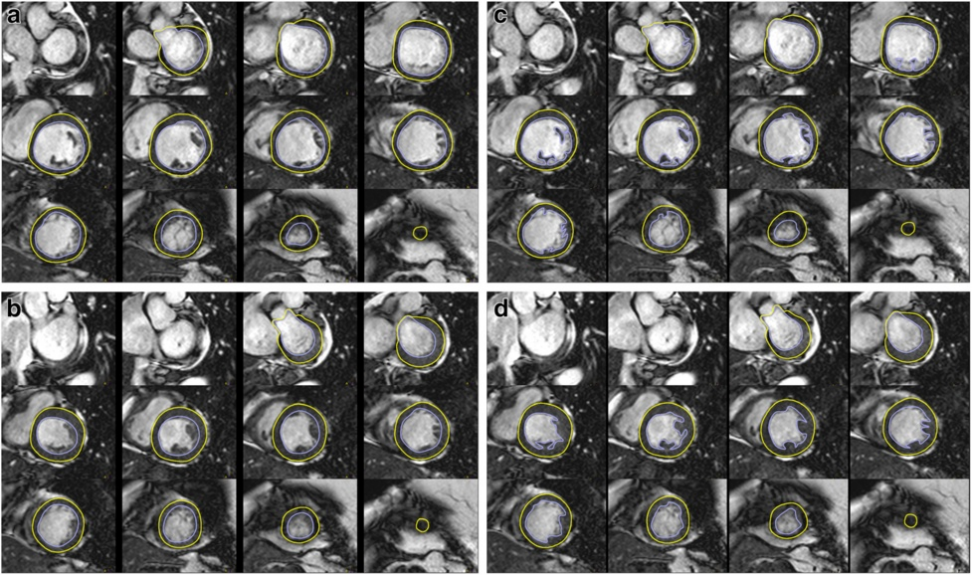
**Left ventricular (LV) chamber quantification.** For LV chamber quantification, the endocardial (blue) and epicardial (yellow) contours are delineated in diastole (top) and systole (bottom) in a stack of short axis slices that cover the whole left ventricle. **a**) and **b**) Illustrates the approach with inclusion of the papillary muscles as part of the LV volume. **c**) and **d**) Shows the approach with exclusion of the papillary muscles from the LV volume.

a) General recommendations

i. Calculated parameters: LV end-diastolic volume, LV end-systolic volume, LV ejection fraction, LV stroke volume, cardiac output, LV mass, and body-surface area indexed values of all except ejection fraction. The parameters quantified may vary depending on the clinical need.

ii. Evaluation of the stack of short axis images with computer-aided analysis packages.

iii. Contours of endocardial and epicardial borders at end-diastole and end-systole (Figure 1).

iv. Epicardial borders should be drawn on the middle of the chemical shift artifact line (when present).

v. The LV end-diastolic image should be chosen as the image with the largest LV blood volume. For its identification, the full image stack has to be evaluated and one phase has to be identified as end-diastole for all the short axis locations.

vi. The LV end-systolic image should be chosen as the image with the smallest LV blood volume. For its identification, the full image stack has to be evaluated and one phase has to be identified as end-systole for all the short axis locations.

vii. Deviations may occur and extra care should be taken in the setting of LV dyssynchrony or severe mitral regurgitation. Aortic valve closure defines end-systole.

viii. Automatic contour delineation algorithms must be checked for appropriateness by the reader.

b) LV volumes

i) Papillary muscles are myocardial tissue and thus ideally should be included with the myocardium. Because not all evaluation tools allow for their inclusion without manual drawing of contours, they are however often included in the volume in clinical practice, which is acceptable. Reference ranges that use the same approach should be used and the inclusion or exclusion of papillary muscles should be mentioned in the report (Figure 1) [8-10].

ii) Outflow tract: The LV outflow tract is included as part of the LV blood volume. When aortic valve cusps are identified on the basal slice(s) the contour is drawn to include the outflow tract to the level of the aortic valve cusps.

iii) Basal descent: As a result of systolic motion of the mitral valve toward the apex (basal descent) care must be taken with the one or two most basal slices. A slice that contains blood volume at end-diastole may include only left atrium (LA) without LV blood volume at end-systole. The LA can be identified when less than 50% of the blood volume is surrounded by myocardium and the blood volume cavity is seen to be expanding during systole. Some software packages automatically adjust for systolic atrioventricular ring descent using cross-referencing from long-axis locations.

c) LV mass

i) Calculation: difference between the total epicardial volume (sum of epicardial cross-sectional areas multiplied by the sum of the slice thickness and interslice gap) minus the total endocardial volume (sum of endocardial cross-sectional areas multiplied by the sum of the slice thickness and interslice gap), which is then multiplied by the specific density of myocardium (1.05 g/ml).

ii) Papillary muscles: Papillary muscles are myocardial tissue and thus ideally should be included with the myocardium, and this is particularly relevant in diseases with LV hypertrophy. However, readers may decide to exclude trabecular tissue and papillary muscles from the myocardial mass. Reference ranges that use the same approach should be used and the inclusion / exclusion of papillary should be specifically mentioned in the report (Figure 1) [8-10].

iii) Basal descent and apex: When the most basal slice contains only a small crescent of basal lateral myocardium and no discernable ventricular blood pool, an epicardial contour for the visible myocardium is included for LV mass only. Similarly, when the most apical slice contains only a circle of myocardium without cavitary blood pool, an epicardial contour without an endocardial contour should be drawn for LV mass calculations.

d) Rapid quantitative analysis

i) A rapid quantitative analysis can also be performed using rotational long axis views (e.g. 2- and 4-chamber views). In cases without expected significant regional variation of wall motion, this technique allows for faster evaluation and is not limited by problems related to basal descent. When the area-length method is used, with either a single long-axis view or a bi-plane approach, specific mention of the analysis technique should be made in the report.

ii) Calculation [11-13]:

– Single long-axis equation: LV volume = 0.85 × (LV-area)^2^/ LV-length. This is typically performed using a 4-chamber view with calculations of LV volume obtained on both end-diastolic and end-systolic phases. LV-area is the planimetered area of the LV cavity from an endocardial contour with the base drawn as a straight line through the medial and lateral aspects of the mitral annulus. LV-length is the linear dimension from the midpoint of the mitral annular line to the apical tip of the endocardial contour.

– Bi-plane equation: LV volume = 0.85 × (LV-area1 x LV-area2)/ LV-length. Here, both 4-chamber (LV-area1) and 2-chamber [or vertical] (LV-area2) long axis views are used to calculate both end-diastolic and end-systolic volumes, similar to the single long-axis equation.

e) Cavity diameter and LV wall thickness can be obtained similar to echocardiography using two CMR approaches [12,14]:

i) Basal short axis slice: immediately basal to the tips of the papillary muscles;

ii) 3-chamber view: in the LV minor axis plane at the mitral chordae level basal to the tips of the papillary muscles.

iii) Both approaches have good reproducibility. The 3-chamber view is most comparable to data obtained with echocardiography.

f) Research:

i) Quantitative evaluation of LV dynamics (e.g. strain, rotation, time-to-peak velocity) is feasible by several imaging techniques (e.g. tagging, DENSE, tissue phase mapping, cine) and requires specific post-processing software. As research applications are evolving and consensus evidence is being accumulated, the Task Force chooses to refrain from making a dedicated statement at this time.

## Right ventricular chamber assessment

### 1. Visual analysis

a) Before analyzing the details, review all cines in cine mode, validate observations from one plane with the others, and check for artifacts and reliability

b) Assessment of global and regional right ventricular (RV) function (septal wall, free wall), where appropriate. Categorization should be noted as: hyperkinetic, normokinetic, hypokinetic, akinetic, dyskinetic.

c) Assessment of both ventricular chambers for hemodynamic interaction (i.e. constrictive physiology)

### 2. Quantitative analysis

**Figure 2:**
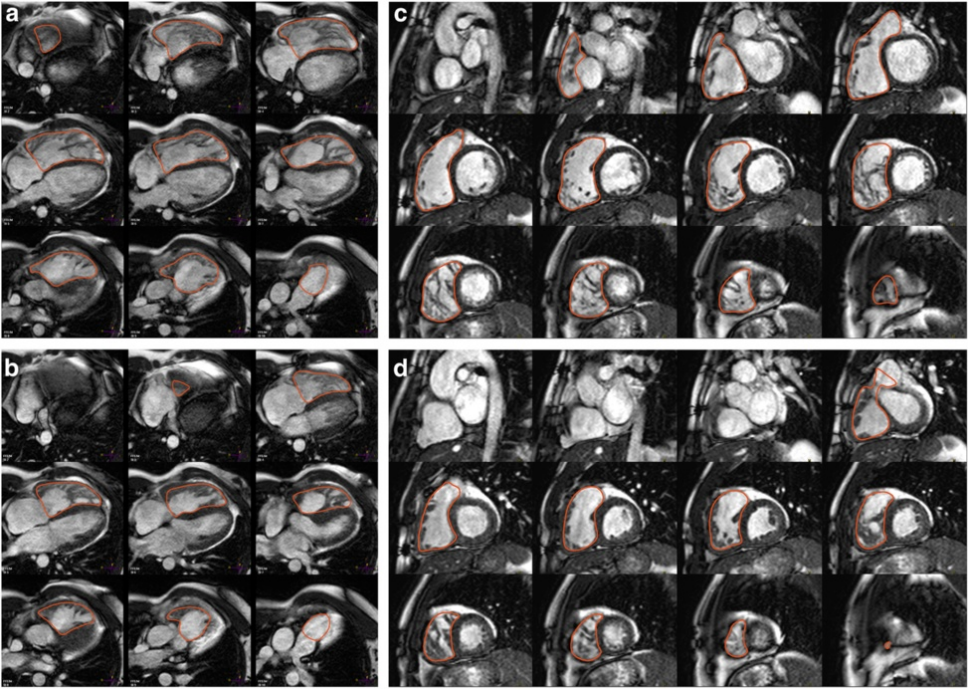
**Right ventricular (RV) chamber quantification.** For RV volume quantification, the endocardial (red) contours are delineated in diastole (top) and systole (bottom) in a stack of transaxial (**a** and **b**) or short-axis (**c** and **d**) slices that cover the whole RV.

a) General recommendations

i) Calculated parameters: RV end-diastolic volume, RV end-systolic volume, RV ejection fraction, RV stroke volume, cardiac output, and body-surface area indexed values of all except ejection fraction. Similar to the LV, the parameters quantified may vary depending on the clinical need [15].

ii) The contiguous stack of short axis images or transaxial cine images is evaluated with computer-aided analysis packages (Figure 2) [16,17].

iii) Transaxial stack of cines covering the RV enable best identification of the tricuspid valve plane.

iv) Endocardial borders are contoured at end-diastole and end-systole (Figure 2).

v) The RV end-diastolic image should be chosen as the image with the largest RV blood volume. For its identification, the full image stack has to be evaluated and one phase has to be identified as end-diastole for all short / transaxial locations.

vi) RV end-systolic image should be chosen as the image with the smallest RV blood volume. For its identification, the full image stack has to be evaluated and one phase has to be identified as end-systole for all short / transaxial locations.

vii) As for the LV, it may be necessary to review all image slices in the stack to define end-systole.

viii) The pulmonary valve may be visualized, and contours are included just up to, but not superior to this level.

ix) Trabeculations of the RV are ignored and a smooth endocardial border is drawn to improve reader reproducibility.

b) RV volumes

i) Total volumes are taken as the sum of volumes from individual 2D slices, accounting for any interslice gap and slice thickness. RV trabeculae and papillary muscles are typically included in RV volumes.

c) RV mass

i) Usually not quantified in routine assessment.

d) Confirmation of results

i) If no intra- or extracardiac shunts are present, the RV and LV stroke volumes should be nearly equal (small differences are seen as a result of bronchial artery supply). Since the LV stroke volume is more reliably determined than the RV stroke volume, the LV data can be used to validate RV data.

## Post processing of myocardial perfusion imaging

### 1. Visual analysis

a) For most clinical indications, visual analysis of myocardial perfusion CMR images is appropriate

b) Work-flow:

i) Display rest and stress perfusion images side-by-side. If possible also display corresponding LGE images.

ii) Adjust window and level: The aim of image adjustment is to set a maximal window width without “overspilling” of the LV cavity signal into the myocardium. Ensure that myocardium before contrast arrival is nearly black and that signal in the RV and LV cavities is bright grey rather than a pure white. Correct level and window settings may require review of both pre- and peak contrast images.

iii) Apply the same contrast, brightness and window settings to all images of the dynamic series.

iv) Review series as cines and/or by scrolling through individual images. Some software packages allow the display of only dynamic images during the first myocardial passage.

v) The key diagnostic feature is contrast arrival and first passage through the LV myocardium.

vi) Visual analysis allows a comparison between regions to identify relative hypoperfusion. Comparison can be made between endocardial and epicardial regions, between segments of the same slice or between slices.

**Figure 3:**
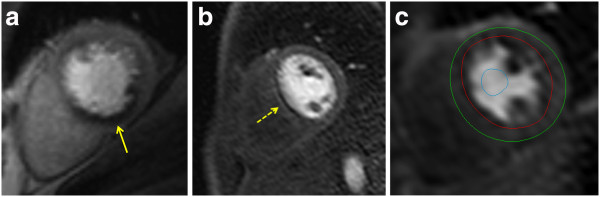
**Perfusion imaging. a**) Perfusion defect in the inferior segments (yellow arrow). Note defect is predominantly subendocardial, has a physiologically credible distribution (right coronary artery territory) and is more than one pixel wide. **b**) Dark banding artifact (yellow arrow). Note defect is very dark, occurs already before contrast reaches the myocardium, is seen in the phase encoding direction (right-left in this case), and is approximately one pixel wide. **c**) Positioning of endocardial (red) and epicardial (green) contours and a ROI in the LV blood pool (blue) for semi-quantitative or quantitative analysis of perfusion data.

c) Compare rest and stress images to identify inducible perfusion defects and artifacts. Note that unlike nuclear perfusion methods, in CMR the finding of a “fixed perfusion defect” on rest and stress perfusion images is not the preferred method to identify myocardial scar. Instead, scar should be identified from LGE images.

d) Criteria for an inducible perfusion defect (Figure 3a):

i) Occurs first when contrast arrives in LV myocardium

ii) Persists beyond peak myocardial enhancement and for several RR intervals (usually >4)

iii) Is more than one pixel wide

iv) Is usually most prominent in the subendocardial portion of the myocardium

v) Often manifests as a transmural gradient across the wall thickness of the segment involved: densest in the endocardium and gradually becoming less dense towards the epicardium

vi) Over time, defect regresses towards the subendocardium

vii) Is present at stress but not at rest

viii) Conforms to the distribution territory of one or more coronary arteries.

e) Interpret location and extent of inducible perfusion defect(s) using AHA segment model [2,7].

i) Estimate number of segments involved

ii) Comment on transmurality of perfusion defect

iii) Indicate extent of perfusion defect relative to scar on LGE

f) Pitfalls of visual analysis

i) Dark banding artifacts (Figure 3b): A common source of false-positive reports are subendocardial dark banding artifacts [18]. These artifacts

– typically occur first and are most prominent when contrast arrives in the LV blood pool, i.e. *before* contrast arrival in the LV myocardium, depending on applied sequence

– lead to a *reduction* in signal compared with baseline myocardial signal (whereas a true perfusion defect will always show an increase in signal compared with the baseline even if this increase is small). These subtle differences can be hard to appreciate visually. It can therefore be helpful to draw a region of interest (ROI) around the suspected artifact and display its signal-intensity-time profile.

– persist only transiently before the peak myocardial contrast enhancement, often for less than approximately 6 RR intervals

– appear predominantly in the phase-encoding direction

– are approximately one pixel wide

Dark banding present at stress and at rest with no corresponding scar on LGE images is also indicative of an artifact [19]. Note however that differences in heart rate and baseline contrast can change the appearance and presence of dark banding between stress and rest perfusion images. Absence of dark banding at rest with typical dark banding at stress should therefore *not* on its own be considered diagnostic for an inducible perfusion defect.

ii) Multi-vessel disease: as visual analysis is a relative assessment of perfusion within an imaged section of the heart, the presence of balanced multi-vessel disease can result in most or all of the imaged section appearing hypoperfused. This can lead to false-negative readings and needs to be considered in relevant clinical circumstances. On visual analysis, a clear endocardial to epicardial signal gradient may be seen in multi-vessel disease [20]. Quantitative analysis of the dynamic perfusion data may be of further help to detect globally reduced myocardial perfusion reserve in multi-vessel disease.

iii) Microvascular disease: Diseases that affect the myocardial microvasculature (e.g. diabetes mellitus, systemic hypertension) may lead to concentric reduction in perfusion [21-24]. This can lead to apparent false-positive readings relative to angiographic methods and needs to be considered in relevant clinical circumstances. Features suggesting microvascular disease are the presence of concentric LV hypertrophy and a concentric, often subendocardial perfusion defect crossing coronary territories. Differentiation from multi-vessel disease can be challenging.

iv) When performing a stress first-rest second perfusion protocol, caution is needed when a defect over a region of infarction may be misinterpreted as reversible. Contrasting the extent and location of reversibility by perfusion imaging with LGE is important to avoid over-calling of segmental ischemia [25].

v) If vasodilator stress during data acquisition was inadequate, visual analysis may lead to false-negative interpretation [26]. Quantitative analysis of the dynamic perfusion data may be of further help to detect globally reduced myocardial perfusion reserve in case of inadequate vasodilator stress.

vi) Signal intensity may vary depending on the distance of myocardium from the surface coil and may lead to misinterpretation if not considered in the analysis. These problems are less likely if acquisition is corrected for coil sensitivity.

### 2. Research tools / Quantitative analysis

a) Objective description of SI change in myocardial perfusion CMR studies can be performed. Several methods have been described for this purpose. In clinical practice, these are rarely required, but they may supplement visual analysis for example in suspected multi-vessel disease or suspected inadequate response to vasodilator stress. Quantitative analysis is also frequently used in research studies.

b) Requirements:

i) Validation and definition of a normal range with the specific pulse sequence and contrast regime used for data acquisition. If only a comparison between regions of the same study is made, establishing a normal range is less relevant.

ii) Consideration of contrast dosage at time of acquisition (high doses are more likely to lead to saturation effects in particular of the arterial input function).

c) Semi-quantitative analysis:

i) Analysis methods that describe characteristics of the SI profile of myocardial perfusion CMR studies without estimating myocardial blood flow are typically referred to as “semi-quantitative analysis methods”.

ii) Work-flow:

– Select an image from the dynamic series with good contrast between all cardiac compartments (some post-processing tools generate an average image of the series).

– Outline LV endocardial and epicardial contours on this image (manual or automated) (Figure 3c).

– Propagate contours to all other dynamic images.

– Avoid myocardial fat in the ROI

– Correct contour position for through-plane motion (some analysis packages register images prior to contours being outlined).

– Depending on the type of analysis to be performed, place a separate ROI in the LV blood pool. Preferably the basal slice is used. Exclude papillary muscles from the ROI.

– Select a reference point in the LV myocardium for segmentation(usually the RV insertion point).

– Segment LV myocardium according to AHA classification [7].

– Generate SI / time profiles for myocardial segments +/- LV blood pool.

– Consider generating division into endocardial and epicardial layers and repeat analysis.

iii) Frequently used semi-quantitative analysis methods (see [27] for detailed review):

– Maximal upslope of the myocardial SI profile, may be normalized to LV upslope [28]

– Time to peak SI of the myocardial SI profile [29]

– Ratio of stress/rest ratios for the above (often referred to as “myocardial perfusion reserve index”) [30]

– The upslope integral (area under the signal intensity-time curve) [31].

iv) Limitations of semi-quantitative analysis methods:

– SI may vary according to distance from coil

– No absolute measurement of myocardial blood flow derived

d) Quantitative analysis

i) Analysis methods that process the SI profile of myocardial perfusion CMR studies to derive estimates of myocardial blood flow are typically referred to as “quantitative analysis methods”. See [27] for review.

ii) Requirements:

– It is a prerequisite for reliable quantification that data acquisition used an appropriate pulse sequence and contrast regime.

– An “input function” for analysis of the myocardial tissue response can be derived from the ROI in the LV blood pool. In order to reduce saturation effects in the blood pool, a “dual-bolus” myocardial perfusion regime may be used, in which the input function is derived from a pre-bolus with a small contrast agent concentration.

– The contrast dose and delivery need to be chosen to minimize saturation effects also the myocardium and typically contrast doses required for reliable quantitative analysis are lower than those optimized for visual analysis in clinical routine.

– A temporal resolution of 1-2 RR intervals is required.

iii) Work-flow:

– Typically, the same source data as for semi-quantitative analysis are used

– Further post-processing may then take place on the same or a separate off-line workstation.

iv) Several analysis methods have been described, including:

– Model-based methods [32]

– Model-independent methods [33]

## Post processing of late gadolinium enhancement studies

### 1. Visual assessment

**Figure 4:**
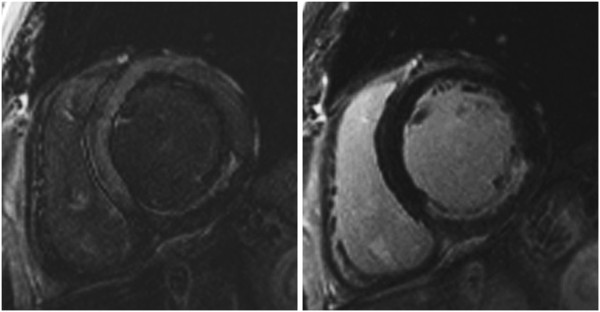
**Late enhancement imaging.** Role of inversion time in late enhancement imaging: On the left panel, normal myocardium has a faint “etched” appearance (darkest at the border with higher image intensity centrally) signifying an inversion time that was set too short and which will lead to underestimation of LGE. On the right panel, the image was repeated with a longer inversion time and demonstrates a larger LGE zone in the inferior wall. Always use the longest inversion time possible that still nulls normal myocardium.

a) For most clinical indications, visual assessment of LGE images is sufficient

b) Work-flow:

i) Modify image window and level so that:

– Noise is still detectable (nulled myocardium should not be a single image intensity)

– LGE regions are not clipped (LGE regions should not be a single image intensity)

ii) Note if normal myocardium has a faint “etched” appearance (darkest at the border with slightly higher image intensity centrally), this signifies an inversion time that was set too short and will lead to underestimation of the true extent of LGE (Figure 4). In general, an inversion time that is slightly too long is preferred to one that is slightly too short [34].

c) Criteria for presence of LGE

i) High SI area that may be as bright as the LV blood pool

ii) Rule out artifacts (see below)

d) Assess pattern of LGE

i) Coronary artery disease (CAD) type: Should involve the subendocardium and be consistent with a coronary artery perfusion territory

ii) Non-CAD-type: Usually spares the subendocardium and is limited to the mid-wall or epicardium, although non-CAD-type should be considered if subendocardial involvement is global [35,36].

e) Interpret location and extent using AHA 17-segment model [7].

i) Comparison of LGE images should be made with cine and perfusion images (if the latter are obtained) to correctly categorize ischemia and viability [25]

ii) Estimate average transmural extent of LGE within each segment (0%, 1-25%, 26-50%, 51-75%, 76-100%) [34].

iii) In patients with acute myocardial infarction, include subendocardial and mid-myocardial hypoenhanced, no-reflow zones as part of infarct size

f) Pitfalls

i) Check for artifacts

– Verify regions with LGE in at least one other orthogonal plane and/or in the same plane obtain a second image after changing the direction of readout

– Bright ghosting artifacts can result from poor ECG gating, poor breath-holding, and long T1 species in the imaging plane (e.g. cerebrospinal fluid, pleural effusion, gastric fluid, etc.) [37]

ii) On non-PSIR (phase sensitive inversion recovery) images, tissue with long T1 (regions below the zero-crossing) may appear enhanced [34,38].

iii) Occasionally, it can be difficult to distinguish no-reflow zones or mural thrombus from viable myocardium. Post-contrast cine imaging may be helpful in this regard.

iv) In case of reduced contrast, the interpretation of additional sequences may be necessary.

### 2. Quantitative analysis

a) Quantitative analysis is primarily performed to measure LGE extent and/or “grey-zone” extent for research purposes. Subjective visual assessment is still a prerequisite to identify poor nulling, artifacts, no-reflow zones, etc, and to draw endocardial and epicardial borders.

b) Multiple different methods of delineating LGE extent are described in the literature, including: manual planimetry, the “n”-SD technique, and the full width half maximum (FWHM) technique (see 3) [39-42].

c) As the research applications are evolving and consensus evidence is being accumulated, the Task Force chooses to refrain from making a dedicated statement at this time regarding the optimal method for quantitative assessment

### 3. Research tools / Quantitative analysis

a) Quantification of LGE extent:

i) Manual planimetry:

– Outline endocardial and epicardial borders

– Manual planimetry of LGE regions in each slice

– Summation of LGE areas

– Multiplication of total LGE area with slice thickness plus interslice gap as well as specific gravity of myocardium provides the approximate LGE weight, which can be used to calculate the ratio of LGE to normal myocardium

– considered subjective

ii) The “n”-SD technique:

– Outlining of endocardial and epicardial borders for the myocardial ROI.

– Selection of a normal “remote” (dark) region ROI within the myocardium to define the reference SI (mean and standard deviation, SD). This subjective approach can affect measurements.

– Is susceptible to spatial variations in surface coil sensitivity.

– Selection of a threshold between normal myocardium and LGE. The relative SNR of scar tissue versus normal myocardium can vary dependent on contrast agent type, dose and time after injection, field strength, type of sequence and other variables including the underlying injury itself. As such, there is no cutoff value, which works for all situations and usually manual tracing is performed as the standard of truth. But (semi-)automated thresholding may improve reproducibility after adequate standardization. As a starting point for semiautomatic thresholding we recommend n+5SD for infarction and n+3SD for myocarditis.

– The presence of LGE within the myocardium is then determined automatically

– requires manual corrections to include no-reflow zones and to exclude artifacts and LV blood pool (errors in the endocardial contour)

iii) FWHM technique:

– Outlining of endocardial and epicardial borders for the myocardial ROI

– Uses the full width of the myocardial ROI SI histogram at half the maximal signal within the scar as the threshold between normal myocardium and LGE

– Determination whether LGE is present or not, and, if LGE is present, selection of a ROI that includes the “maximum” signal. This subjective selection can affect measurements.

– Is also susceptible to spatial variations in surface coil sensitivity, albeit perhaps less so than the “n”-SD technique [40].

– Considered more reproducible than the n-SD technique [42].

– Since technique assumes a bright LGE core, may be less accurate than the “n”-SD technique if LGE is patchy or grey [43]

– Requires manual corrections to include no-reflow zones and to exclude artifacts and LV blood pool (errors in the endocardial contour)

b) Peri-infarct" zone: [44,45]

– multiple methods of quantifying the extent of grey zones are reported.

– The Task Force is not able to provide a dedicated statement at this time due to a lack of consensus available from the published literature.

– can account for spatial variations in coil sensitivity

c) T1 mapping: [46-49]

– may be helpful in identifying diffuse myocardial fibrosis

– may provide a quantitative assessment of the extent of fibrosis

– multiple sequences and imaging protocols are described in the literature

– as the research application(s) are evolving and consensus evidence is being accumulated, the Task Force chooses to refrain from making a specific recommendation at this time

## Post processing of T2-weighted imaging

### 1. Visual analysis

**Figure 5:**
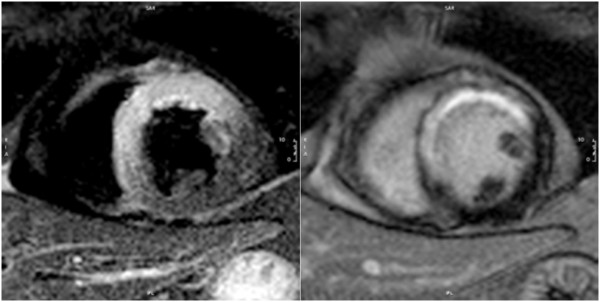
**CMR in acute myocardial infarction.** Acute reperfused infarction of the left anterior descending artery territory. Left: T2-weighted image (short-tau inversion recovery, STIR) in a midventricular short axis view with increased SI in the affected segments. Right: LGE image in the same orientation.

a) The visual analysis should aim for detecting or excluding regions with significant SI increase, indicating increased free water content.

b) Qualitative, visual analysis of myocardial SI may be sufficient for diseases with regional injury to the myocardium such as acute coronary syndromes/infarction (Figure 5), early stages of myocarditis and sarcoidosis.

c) Work-flow:

i) Identify and display appropriate image(s)

ii) Modify image contrast and brightness to minimize SI in a background noise area (noise should still be detectable) and to reduce the maximal SI displayed to an area with the highest SI without allowing for “over-shining” with erroneous display of pixels as white

iii) Check for artifacts

d) Criteria for edema:

i) Clearly detectable high SI area

ii) Respecting anatomical borders

iii) Following an expected regional distribution pattern (mainly subendocardial, transmural, mainly subepicardial, focal)

iv) Verifiable in two perpendicular views

e) High SI areas suggestive of myocardial edema should be compared to

i) regional function

ii) other tissue pathology such as irreversible injury: scar/fibrosis, infiltration

f) Pitfalls of visual analysis:

i) Surface coil reception field inhomogeneity: The uneven distribution of the sensitivity of the receiver in surface coil may lead to false low SI in segments most distant to the coil surface or false high SI in segments closest to the coil surface, especially in dark-blood triple-inversion recovery spin echo (STIR, TIRM) images. Therefore, the body coil or a reliable and accurate correction algorithm should be used to ensure a homogeneous signal reception.

ii) Low SI artifacts: Arrhythmia or through-plane motion of myocardium may cause artifacts, making large areas appear with false low SI, especially in dark-blood triple-inversion recovery spin echo images.

iii) High SI artifacts: In dark-blood triple-inversion recovery spin echo images, slow flowing blood may lead to insufficient flow suppression and results in high SI, which may be confused with myocardial edema.

### 2. Semi-quantitative analysis

a) Because low SI artifacts can lead to SI distribution patterns similar to extensive myocardial edema, a mere visual analysis may lead to incorrect results. SI quantification with reference regions is much less sensitive to these errors and therefore is recommended.

b) Requirements:

i) Tested normal values for SI values or ratios.

c) Work-flow

i) Global SI analysis:

– Outline LV endocardial and epicardial contours.

– For the T2 SI ratio, draw the contour for a ROI in a large area of the skeletal muscle closest to the heart and to the center of the reception field of the coil (for short axis views preferably in the M. serratus anterior).

ii) Regional SI analysis:

– Draw the contour for a ROI in the affected area and divide the SI by that of the skeletal muscle.

iii) While a cut-off of 1.9 can be used for dark-blood triple-inversion recovery spin echo [50], a locally established value is recommended, because SI and ratio values may vary between sequence settings (especially echo time (TE)) and scanner models. For these images, a color-coded map, based on the parametric calculation and display of myocardial pixels with a SI ratio of 2 or higher, can also be used.

### 3. Quantitative analysis

a) Research areas / advanced imaging

i) T2 mapping [51]:

– can account for spatial variations in coil sensitivity

– multiple sequences and imaging protocols are described in the literature

– may provide quantitative measurements of edema

– as research application(s) are evolving and consensus evidence is being accumulated, the Task Force chooses to refrain from making a dedicated statement at this time

## Post processing of T2* imaging

### 1. Visual analysis

T2* imaging always requires a quantitative analysis. Visual analysis is used to ensure optimal image quality, which is the most important factor in the accuracy of data analysis.

### 2. Quantitative analysis

a) Evaluation of T2* always requires a quantitative analysis using software with regulatory approval for T2* evaluation in patients

b) Full thickness ROI defined of the ventricular septum

i) Take care to avoid blood pool and proximal blood vessels

ii) Septal ROI avoids susceptibility artifact from tissue interfaces

**Figure 6:**
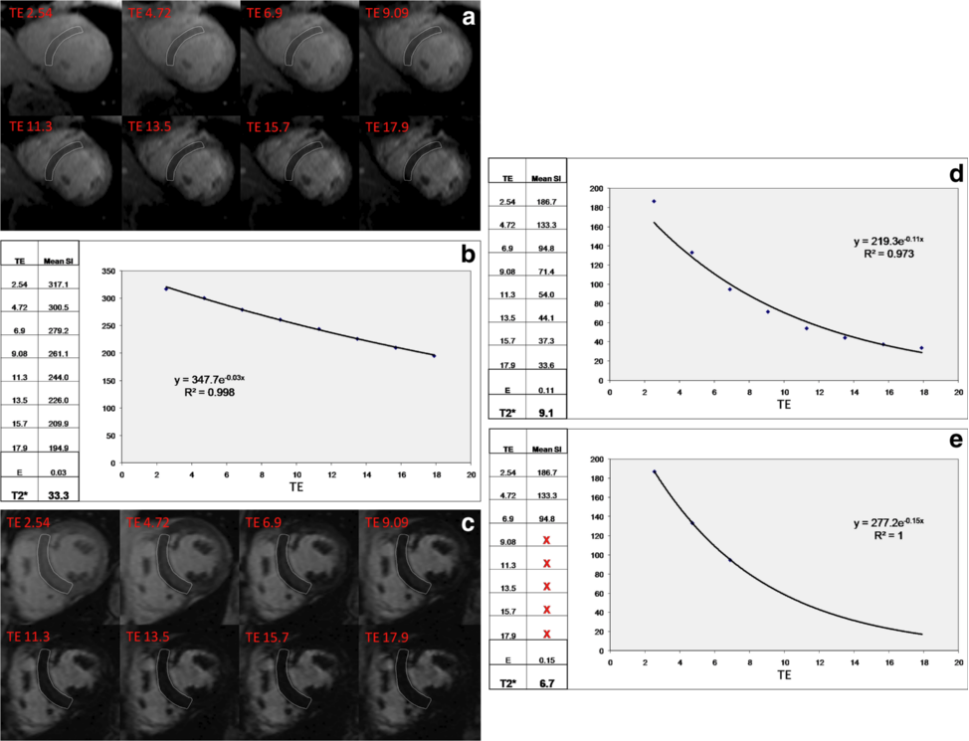
T2* imaging to assess myocardial iron overload. a) T2* scan of a normal heart showing slow signal loss with increasing TE. **b**) Decay curve for normal heart. T2* = 33.3ms. **c**) Heavily iron overloaded heart. Note there is substantial signal loss at TE = 9.09. **d**) Decay curve for heavily iron overloaded heart showing rapid signal loss with increasing TE. The curve plateaus as myocardial SI falls below background noise. **e**) Values for higher TEs are removed (truncation method) resulting in a better curve fit and a lower T2* value.

c) Mean myocardial SI from the ROI is plotted against TE (Figure 6)

i) SI falls with increasing TE

ii) A mono-exponential curve is fitted to the data

iii) The time for the decay of SI falls (shorter T2*) with increasing iron burden

iv) In heavily iron overloaded patients, SI for higher TEs may fall below background noise causing the curve to plateau and underestimating T2*.

v) This can be compensated for by:

– Truncating the curve by removing later echo times (Figure 6e) [52,53]

– This issue is not significant when using the double inversion recovery (black blood) sequence [54]

d) Cut-off values (at 1.5 Tesla):

i) Normal cardiac T2* is 40ms [55]

ii) T2* < 20 ms = cardiac iron overload [56]

iii) T2* <10ms indicates increased risk of development of heart failure [57]

e) CMR assessment of T2* at 3T for assessment of iron overload cardiomyopathy cannot be recommended at this time. T2* shortens with increasing field strength making assessment of severe iron overload more problematic, and there is a lack of clinical verification.

## Flow image interpretation and post processing

### 1. Visual analysis

**Figure 7:**
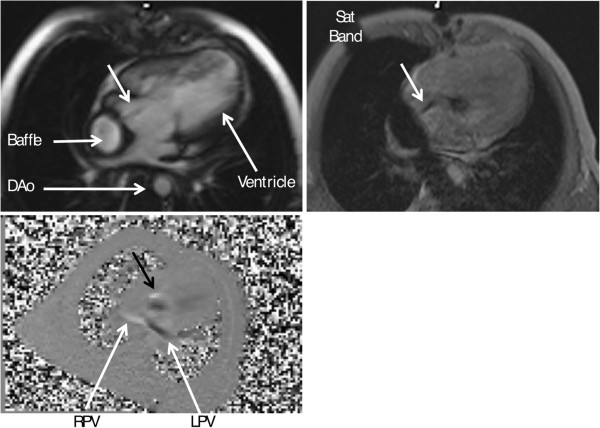
**Flow imaging in congenital heart disease.** Visualizing flow across a fenestration in a single ventricle after Fontan. Upper left is the magnitude image, upper right is the gradient echo image with a saturation band and lower left image is an inplane velocity map in the 3-chamber view demonstrating the fenestration flow (black arrow). Note opposite directions of the flow on the inplane velocity map in right (RPV, white flow) and left pulmonary veins (LPV, black flow). DAo - descending aorta.

a) Appropriately aligned acquisitions of cines and stacks of cines can give valuable information on flow in relation to adjacent structures, notably on the directions, time courses and approximate dimensions of jets resulting from valve regurgitation, stenoses or shunts. Such information can be important in assessing the credibility of measurements of flow, which may be subject to several possible sources of error. Gradient echo cines differ somewhat from SSFP in terms of degrees of signal augmentation or reduction attributable to flow effects. Of note, SSFP can provide clear delineation between the relatively bright signal from voxels aligned within the coherent core of a jet, and low signal from the shear layer that bounds such a jet core. In- or through-plane phase contrast flow velocity acquisitions can also provide visual information on the directions, dimensions and time courses of flow; it can also image morphology, which can yield a clue to the etiology of an abnormal jet (e.g. imaging accelerated flow jet of a coarctation or of valve insufficiency, or flow direction across an atrial septal defect or Fontan fenestration (Figure 7)) [58,59].

b) Pitfalls:

i) Flow appearances on both cine and phase encoded acquisitions are highly dependent on image location and orientation, especially in the case of jet flow

ii) If the range of velocity encoding (VENC) is set too high, visualization of the jet may not be obtained. If it is set too low, a mosaic pattern on the images will be visualized [60].

iii) If slice thickness is too large on in-plane velocity mapping, the higher velocities will be “averaged out” with the lower velocities and stationary tissue; jets and flow may not be visualized correctly.

iv) If the annulus of valves is very dynamic or the imaging plane is not set correctly, the valve morphology may not be visualized.

v) If imaging in the presence of metal containing devices, signal loss may be present as artifact and interpretation must proceed with caution.

vi) The TE should be as low as possible for increased accuracy, especially with high velocity turbulent jets; this should be kept to 3.5 ms or lower [61].

### 2. Quantitative analysis

**Figure 8:**
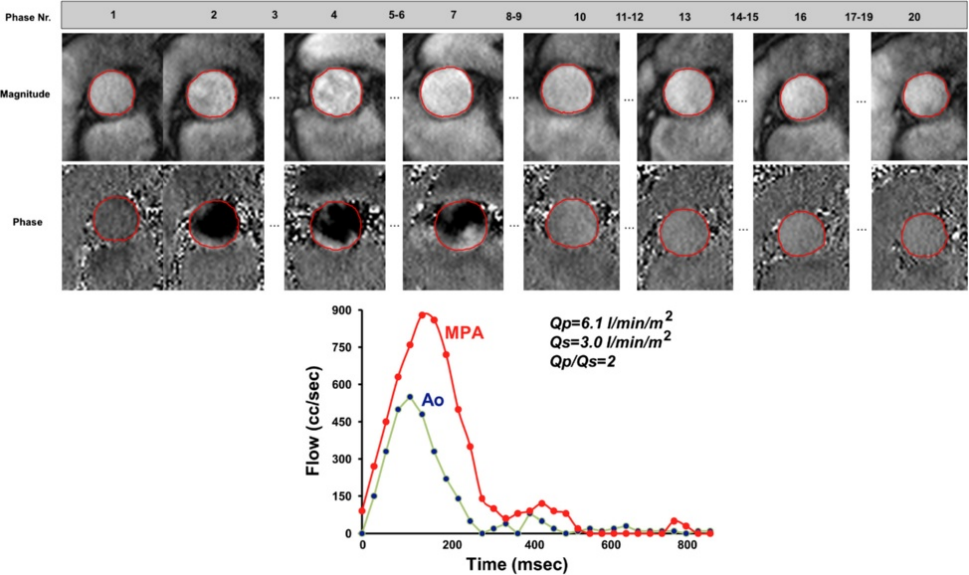
**Quantification of blood flow. (top)** Contours were drawn delineating the aortic lumen at the sinotubular level during all 20 phases of the cardiac cycle to assess aortic flow. **(bottom)** Flow curves from measurements in the ascending aorta and in the pulmonary artery in a patient with ventricular septal defect showing a left-to-right shunt.

a) Work-flow:

i) Load phase and magnitude images into software. Window the magnitude and phase images to the appropriate brightness and contrast so that the borders of the ROI are sharp.

ii) Examine the images to ensure the quality is sufficient and that the VENC was not exceeded, or there was little contrast (ie the VENC was too high).

iii) Trace the borders of the vessel of interest on each phase and magnitude image so that only the cavity of the vessel is included (Figure 8); make sure the noise outside the vessel is not included. Check that this is performed correctly on the magnitude images always keeping in mind that it is the phase images that contain the encoded information.

iv) Baseline-correction or comparison to a phantom for flow measurements may be considered [62,63].

v) Directly calculated parameters include: antegrade volume, retrograde volume, peak velocity and mean velocity

vi) Derived parameters include:

– Net volume [ml| = antegrade volume - retrograde volume

– Regurgitant fraction [%] = ( retrograde volume / antegrade volume) * 100

– Cardiac output (liters/min = (net volume [ml] x heart rate [beats/minute])/1000) and cardiac index (cardiac output/BSA) when integrating heart rate and body surface area

– Regional flow to both lungs by measuring cardiac output in each branch pulmonary artery (eg percentage of flow to the right lung = (right pulmonary artery flow / right pulmonary artery flow + left pulmonary artery flow) × 100).

– Regurgitant volumes of the atrioventricular valves may be obtained by either of 2 methods: A) direct measurement of diastolic flow across the valve and subtraction of systolic forward flow across the associated semilunar valve or B) measurement of stroke volume using cine CMR and subtraction of forward flow across the associated semilunar valve.

b) Pitfalls:

i) On the phase images, the area of flow may be slightly larger than the area of the magnitude images.

ii) If the VENC is exceeded, most software packages allow for moving/changing the “dynamic range” of the images so that the VENC is not exceeded. For example, if the peak velocity in the aorta is 175 cm/s and the VENC was set at 150 cm/s, the dynamic range is between -150 cm/s and +150 cm/s (i.e. 300 cm/s). This may be moved to -100 cm/s and +200 cm/s to account for this accelerated velocity. This will be demonstrated on the graph of the velocity where the phase in which the VENC is exceeded does not “clip” (appears to go the wrong way) after correction.

iii) In general, the area that exceeds the VENC in the ROI is in the center of the vessel and not at the edges; if at the edges, it is usually (but not always) outside the vessel.

iv) If imaging in the presence of devices, signal loss may be present as artifact and interpretation must proceed with caution.

v) When measuring peak velocity, some software packages will determine the peak velocity in one pixel in the ROI whereas others may take the peak velocity of the average of a few adjacent pixels in the ROI. By reporting the peak velocity in a single pixel, noise may make this measurement inaccurate. By reporting this as an average of a few adjacent pixels, noise is less of an issue, however, the true “peak velocity” may be higher than the reported value. These factors must be kept in mind and interpretation may need to be adapted to the measurement technique used.

vi) When attempting to measure peak velocity using through plane velocity mapping along a vessel, interpretation should be tempered by the notion that this parameter may be an underestimate as the true peak velocity lies somewhere along the vena contracta; the through plane velocity map may not have been obtained at the level of the true peak velocity. If the vena contracta is itself narrow or ill defined, jet velocity mapping is unlikely to be possible

vii) Peak velocity is only minimally affected by small background phase offsets, while volume measurements can be dramatically affected by even a small background phase offset due to the cumulative aspect of integration overspace (within the ROI) and time (over the cardiac cycle). Dilatation of a great vessel tends to increase error of this type [64].

viii) Orientation of the image plane perpendicular to flow direction can have a significant impact on peak velocity measurement, while not significantly affecting volume flow [65].

### 3. Research tools

a) 4D flow: The utility of this approach is the subject of ongoing research.

b) Real time velocity mapping: The utility and post processing algorithm best applied to this approach is the subject of ongoing research.

## Post processing of angiography of thoracic aorta, pulmonary arteries and veins

### 1. Visual analysis

**Figure 9:**
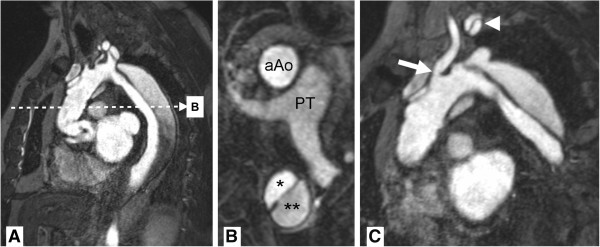
**MR-angiography.** Stanford A aortic dissection after surgical repair with graft of ascending aorta. Panel **A** shows a source image of breath-held 3D gradient recalled echo sequence after contrast injection. Multiplanar reformats in axial orientation (**B**) at the level of the pulmonary trunk (PT) show a normally perfused ascending aorta graft (aAo) and persistent dissection in descending aorta with true (*) and false (**) lumina. Double oblique reformat (**C**) shows narrowing at the origin of the left common carotid artery (arrow) and dissection membrane propagating into the left subclavian artery (arrowhead) with perfusion of both lumina.

a) Maximum intensity projections (MIP) for first review of 3D data and for demonstration purposes (Figure 9A). Volume rendered (VR) techniques may be used for demonstration purposes, but not for detailed analysis.

b) Aorta [67]:

i) Wall thickness: Review balanced steady state free precession (bSSFP) or turbo spin echo images.

ii) Wall irregularities: Review 3D-MRA source images and bSSFP or turbo spin echo.

c) Pulmonary arteries [68]:

i) Multiplanar double oblique and targeted MIP reconstructions for assessment of wall adherent thrombi, wall irregularities and abrupt diameter changes.

d) Pulmonary veins [69]:

i) Screen for atypical insertion and small accessory veins.

e) Coronary arteries:

i) Coronary MRA may play a role in assessment of congenital anomalies but not usually in the context of ischemic heart disease. The course of coronary arteries is best assessed on source images, multiplanar reconstructions (MPR) or targeted MIP reconstructions.

### 2. Quantitative analysis

**Figure 10:**
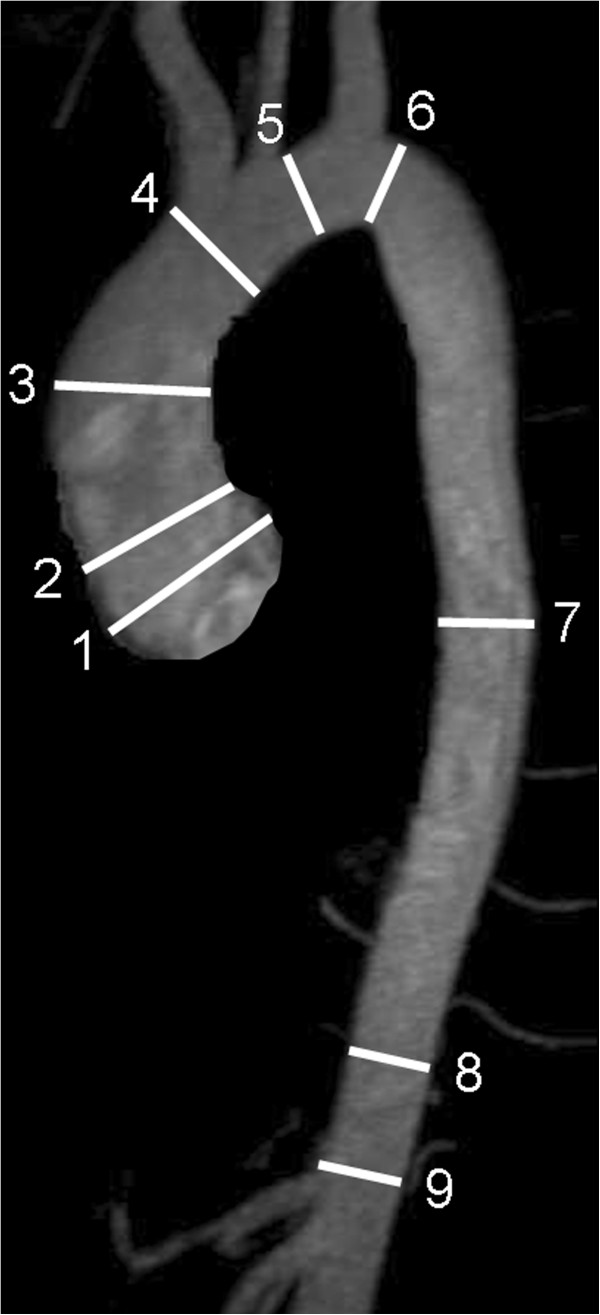
**Anatomic landmarks for standardized reporting of diameters of the aorta at the level of sinuses of valsalva (1), sinotubular junction (2), mid-ascending aorta (3), proximal to brachiocephalic trunk (4), between left common carotid and left subclavian arteries (5), distal to left subclavian artery (6), mid-descending aorta (7), diaphragm (8), abdominal aorta above coeliac trunk (9).** (Adapted from [66]).

a) Aorta:

i) Multilevel measurements of aortic diameters on double oblique multiplanar images perpendicular to blood flow at standardized levels (Figure 10) [66]. Measurements should be obtained at diastole if possible.

ii) Inner diameter (lumen width). In the presence of wall thickening (e.g. thrombus or intramural hematoma) outer diameter including vessel walls should also be reported.

iii) Diameters of sinuses or sinotubular junction may not be measured on ungated images since motion artifacts can lead to blurring and may result in diameter under- or overestimation. These require ECG gated acquisitions, either from 3-dimensional SSFP acquired in late diastole, or from a contiguous stack of cines aligned to transect the axis of the aortic root. Consistent methods of acquisition and measurement are essential for the evaluation of any change over time, for example by the measurement in late diastole, of all three sinus-commissure dimensions of the aortic root, which may dilate asymmetrically.

iv) Standardized report including table of diameters.

b) Pulmonary artery:

i) The widest inner diameter is measured perpendicular to the long axis of the main pulmonary artery at the level of the pulmonary bifurcation in transaxial slices

c) Pulmonary veins:

i) MPR of pulmonary veins perpendicular to blood flow for diameter measurements.

## Abbreviations

bSSFP: Balanced steady state free precession; CAD: Coronary artery disease; CMR: Cardiovascular magnetic resonance; FWHM: Full width half maximum; LGE: Late Gadolinium enhancement; LV: Left ventricle; MIP: Maximum intensity projection; MPR: Multiplanar reconstruction; PSIR: Phase sensitive inversion recovery; ROI: Region of interest; RV: Right ventricle; SD: Standard deviation; SI: Signal intensity; STIR: Short-tau inversion recovery; TE: Echo time; TI: Inversion time; TR: Repetition time; TSE: Turbo spin echo; VENC: Velocity encoding; VR: Volume rendering.

## Competing interests

The authors declare that they have no competing interests.

## Authors’ contributions

JSM wrote paragraphs, edited manuscript, corresponding author. DAB, JB, SDF, MAF, MGF, RJK, FvKB, CMK, DJP, SP and EN wrote paragraphs, edited manuscript. All authors read and approved the final manuscript.
